# DHCR24 Knockdown Induces Tau Hyperphosphorylation at Thr181, Ser199, Ser262, and Ser396 Sites via Activation of the Lipid Raft-Dependent Ras/MEK/ERK Signaling Pathway in C8D1A Astrocytes

**DOI:** 10.1007/s12035-022-02945-w

**Published:** 2022-07-08

**Authors:** Meiting Mai, Xiaorou Guo, Yue Huang, Wenbin Zhang, Yixuan Xu, Ying Zhang, Xiaojing Bai, Junfeng Wu, Hengbing Zu

**Affiliations:** Department of Neurology, Jinshan Hospital Affiliated to Fudan University, No.1508 Long-hang Road, Jinshan district, Shanghai, 201508 China

**Keywords:** DHCR24, Cholesterol, Tau hyperphosphorylation, Astrocyte, Alzheimer’s disease

## Abstract

The synthetase 3β-hydroxysterol-Δ24 reductase (DHCR24) is a key regulator involved in cholesterol synthesis and homeostasis. A growing body of evidence indicates that DHCR24 is downregulated in the brain of various models of Alzheimer’s disease (AD), such as astrocytes isolated from AD mice. For the past decades, astrocytic tau pathology has been found in AD patients, while the origin of phosphorylated tau in astrocytes remains unknown. A previous study suggests that downregulation of DHCR24 is associated with neuronal tau hyperphosphorylation. Herein, the present study is to explore whether DHCR24 deficiency can also affect tau phosphorylation in astrocytes. Here, we showed that DHCR24 knockdown could induce tau hyperphosphorylation at Thr181, Ser199, Thr231, Ser262, and Ser396 sites in C8D1A astrocytes. Meanwhile, we found that DHCR24-silencing cells had reduced the level of free cholesterol in the plasma membrane and intracellular organelles, as well as cholesterol esters. Furthermore, reduced cellular cholesterol level caused a decreased level of the caveolae-associated protein, cavin1, which disrupted lipid rafts/caveolae and activated rafts/caveolae-dependent Ras/MEK/ERK signaling pathway. In contrast, overexpression of DHCR24 prevented the overactivation of Ras/MEK/ERK signaling by increasing cellular cholesterol content, therefore decreasing tau hyperphosphorylation in C8D1A astrocytes. Herein, we firstly found that DHCR24 knockdown can lead to tau hyperphosphorylation in the astrocyte itself by activating lipid raft-dependent Ras/MEK/ERK signaling, which might contribute to the pathogenesis of AD and other degenerative tauopathies.

## Background

Among multiple pathological changes that may contribute to the pathogenesis of Alzheimer’s disease (AD), tau pathology is of a central role. For decades, microtubule-associated protein tau has been viewed to be mainly expressed in neurons. However, it is noteworthy that mRNA and the protein of tau can also be detected in astrocytes, though in a less-abundant level [1–5]. Recently, pathological tau has been identified in astrocytes from AD animal models and AD patients [6, 7]. Besides, tau inclusion in astrocytes also contains hyperphosphorylated and conformation-altered tau [8–11]. Intriguingly, kinases involved in tau hyperphosphorylation, such as ERK, JNK, and GSK3β, were also expressed in tau-bearing astrocytes [6, 12]. Till now, less attention has been paid on the origin of astrocytic tau. Previous studies have not systemically looked into the mechanism of tau hyperphosphorylation in the astrocyte itself. Generally speaking, tau accumulations found in astrocytes have usually been viewed to be internalized from neuronal phosphorylated tau, since tau is mainly expressed by neurons [13]. However, a recent study has shown that injecting non-transgenic mice with pathological tau extracted from AD patients induced tau aggregates only in neurons [14]. Moreover, it has been found that phosphorylated tau released by neurons was not taken up by astrocytes [15]. Therefore, it is possible that the astrocyte itself might produce pathological hyperphosphorylated tau that could contribute to AD and other tauopathies.

DHCR24, also named seladin-1, is a crucial enzyme regulating cholesterol biosynthesis. In the post-lanosterol pathway of cholesterol synthesis, the final step in the Bloch pathway or the first step in the Kandutsch-Russell pathway is catalyzed by the DHCR24 [16, 17]. In the DHCR24-silencing neuronal cell model, it was found that cellular desmosterol level is significantly elevated, and cellular cholesterol content is greatly decreased, suggesting the remarkable effects of DHCR24 expression on cellular cholesterol homeostasis [18, 19]. Interestingly, mounting evidence has suggested that DHCR24 was downregulated in the brain of multiple animal models of AD and patients with dementia, indicating that deficiency of brain cholesterol might contribute to AD pathogenesis [20–25]. In the adult brain, neurons require an external cholesterol supply, which is supplied by astrocytes through ApoE-containing lipoproteins [26]. Intriguingly, it has been found that a number of genes involved in cholesterol synthesis are downregulated in aging astrocytes and astrocytes isolated from AD mice, including DHCR24, which implies the decrease in cholesterol synthesis and cholesterol loss in astroglia cells during AD process [25, 27]. For decades, DHCR24 knockdown has been demonstrated to correlate with apoptosis, inflammation and Aβ pathology by disrupting cellular cholesterol homeostasis [18, 28, 29]. Moreover, in previous study, we found that silencing DHCR24 also induced tau phosphorylation in neurons [19, 30, 31]. As astrocyte is the major cell type in the brain that produces and provides with cholesterol, DHCR24 downregulation found in AD astrocytes might lead to more profound influence in AD pathology in cholesterol-dependent manner. Therefore, it is likely that DHCR24 downregulation in astrocytes might also be associated with astrocytic tau phosphorylation.

Cholesterol has been found to be a major component of the plasma membrane and lipid rafts/caveolae. As an indispensable structural lipid, cholesterol loss could lead to the membrane disorganization and disruption of membrane lipid rafts, resulting in the alterations of lipid raft-dependent cell signaling, such as PI3-K/Akt/GSK3β and MAPK/ERK [19, 32]. Meanwhile, the activation of these signaling was identified to induce tau phosphorylation [33]. We previously found that DHCR24 knockdown promoted tau phosphorylation via regulating lipid rafts/caveolae-dependent signaling in neuronal cells [19, 31]. Those sites are classic sites for hyperphosphorylation of tau in neurons during the AD process and are proven to be pathological when hyperphosphorylated. Whereas, similarities exist between astrocytic phosphorylated tau and neuronal phosphorylated tau, either in phosphorylation sites or associated protein kinases [10–12]. Many sites of neuronal tau protein are also hyperphosphorylated in tau-bearing astrocytes, and might also be pathological [34]. Hence, we hypothesize that reduced DHCR24 expression in astrocytes is likely to be involved in astrocytic tau pathology through dysregulation of cellular cholesterol homeostasis. Herein, in this study, DHCR24 knockdown and DHCR24 knock-in C8D1A astrocyte cell lines were generated by infecting cells with a lentivirus expressing DHCR24-shRNA or DHCR24-cDNA. We explored whether DHCR24 downregulation affects tau hyperphosphorylation in astrocytic tauopathy through the activation of lipid rafts/caveolae-dependent RAS/MEK/ERK signaling, which could contribute to the tauopathy in AD and other neurodegenerative diseases.

## Materials and Methods

### Antibodies and Reagents

DHCR24 (#2033, Cell Signaling Technology), Tau5 (AHB0042, Thermo), Phospho-Tau (Thr181) (ab254409, Abcam), Phospho-Tau (Ser199) (#29,957, Cell Signaling Technology), Phospho-Tau (Ser396) (#9632, Cell Signaling Technology), Actin (#2118, Proteintech), cavin1 (#18,892–1-AP, Proteintech), Phospho-Tau (Ser262)(ab131354, Abcam), Phospho-Tau (Thr231)(#4137, ABclonal), Ras(27H5)(#3339, Cell Signaling Technology), p44/42 MAPK (ERK1/2) (137F5)(#4695S, Cell Signaling Technology), Phospho-p44/42 MAPK (Erk1/2)(Thr202/Tyr204)(#4730S, Cell Signaling Technology), MEK1/2 (D1A5)(#8727, Cell Signaling Technology), Phospho-MEK1/2 (Ser217/221)(#9154, Cell Signaling Technology), U0126 (#9903, Cell Signaling Technology), Propidium Iodide (abs9105, Absin), Methyl-β-cyclodextrin (abs42021762, Absin), Filipin III (abs42018484, Absin), Oil Red O (CAS:1320–06-5, O0625-25G, Sigma), HRP-conjugated Affinipure Goat Anti-Rabbit IgG(H + L)(SA00001-2, Proteintech)**,** HRP-conjugated Affinipure Goat Anti-Mouse IgG (H + L)**(**SA00001-1, Proteintech).

### Cell Culture

The C8D1A cell line was generated by Alliot F and Pessac B in 1984 and was used for the in vitro study of astrocytes [35]. These cell clones are from 8-day postnatal explants, without the addition of either carcinogens or oncogenic viruses. The properties of C8D1A cells are remarkably stable for their morphology and their expression pattern did not change over 100 generations. Besides, these cloned cell lines share characteristics with the main types of astrocytes, such as the expression of symbolized molecules and the morphology. Therefore, in several studies, this cell line has been an acceptable and alternative choice for the study of astrocytes in vitro. C8D1A and HEK293T cells were cultured in 4.5 g/L glucose Dulbecco’s Modified Eagle Medium (DMEM) (Gibco, China) containing 10% fetal bovine serum ((BI, Israel), 100 U/ml penicillin, and 100 μg /ml streptomycin. All cells were cultured at 37 ℃ with 5% CO_2_ in the incubator.

### Lentiviral Construction

Mouse DHCR24 cDNA in pLVX-Puro vector (pLvx-Puro-DHCR24) and empty pLVX-Puro vector (both obtained from GENEWIZ, China) were cloned into lentivirus transfer (HIV) plasmid and packed into third-generation self-inactivating lentiviral particles in 293 T cells. Transfections were performed using LipoD293 (SL100668, SignaGen) following the manufacturer’s instruction. The viral supernatant was collected at 24, 48, and 72 h after transfection and the cell debris was removed by centrifuging at 1000 g for 5 min. The viral supernatant was stored at − 80 ℃ before infecting cells for DHCR24 overexpression. We obtained the DHCR24-shRNA lentivirus from Hanbio Biotechnology (China). The ShRNA target sequence is as follows: (5′- GCGAGGAATTCTGGGAGATGTTCGA -3′).

### Lentivirus-Mediated Gene Transfection in C8D1A Cells

For lentiviral transduction, cells were seeded on a 6‐well plate for 24 h to reach 50% confluence before infection. DHCR24-cDNA, DHCR24-shRNA-containing lentivirus, and their control lentiviral particles were used to infect cells at multiplicity of infection (MOI) 25. The culture medium was added with 10 ug/mL hexadimethrine bromide for optimal infection and was removed after a 24-h culture. After 72 h, a medium containing 2 μg/mL puromycin was used for cell culture to kill noninfected cells. Western blot and quantitative real-time polymerase chain reaction (qPCR) were performed to confirm whether DHCR24 knock-in and knockdown cell lines were successfully established.

### Western Blot

Total protein lysates for Western blot were prepared by lysing the cells using SDS lysis buffer (KeyGen BioTECH, China) supplemented with a protease and phosphatase inhibitor. The lysates were centrifuged at 12,000 × g, 4 ℃ for 15 min. We then used a BCA protein assay kit (KeyGen BioTECH, China) to verify protein concentrations, and protein (20 μg per lane) was loaded into 10% SDS‐PAGE gel and subsequently transferred onto a PVDF membrane (0.25- or 0.45-μm pore, Millipore). After that, the PVDF membrane was blocked in 5% non-fat milk at room temperature (RT) for 1 h. The membranes then were incubated with primary antibodies at 4 ℃ overnight. After incubation with species-specific horseradish peroxidase (HRP)-linked secondary antibodies for 1 h at RT, the proteins were detected using enhanced chemiluminescence substrates (Millipore) and Chemiluminescent (Bio-Rad, Hercules, CA, USA) and analyzed via Tannon 4600 Chemiluminescence Image Analysis System version 3.0 (Tannon, Shanghai, China).

### Immunofluorescence Staining

Cells were seeded on a 12-well plate to reach 50% confluence. All the following procedures were performed at RT unless otherwise noted. Cells were at first fixed in 4% paraformaldehyde (PFA) for 30 min and permeabilized in 0.5% Triton X-100 for 20 min. Then, cells were incubated for 1 h in the blocking solution (Beyotime, China). Afterwards, cells were stained with Phospho-p44/42 MAPK (Erk1/2) (Thr202/Tyr204) (D13.14.4E) (#4730S, 1:200, Cell Signaling Technology) at + 4 °C overnight, followed by staining with anti-rabbit Alexa594 (1:500, Cell Signaling Technology) secondary antibody for 1 h. After three times washes in PBS for 10 min, cell nuclei were stained with 4′,6-diamidino-2-phenylindole (DAPI) (KeyGen BioTECH, China) for 5 min. Eventually, cells were imaged using a fluorescent microscope (Olympus, Tokyo, Japan).

### Filipin III Fluorescence Staining

The staining of Filipin III was conducted according to a previous study [36]. Briefly, cells were seeded on cover-glass bottom dishes for preparation. Cells were incubated with or without methyl-β-cyclodextrin (MβCD) at a final concentration of 10 mM for 30 min at 37 °C. Afterwards, cells were fixed in 4% PFA for 10 min at RT, followed by incubation with a working solution of Filipin III (0.1 mg/ml) for 30 min. After two times of washing with PBS, cells were stained with propidium iodide (PI, 0.35 μg /ml) for 5 min. Eventually, cells were imaged using a confocal laser scanning microscope (405 nm, Leica sp5, Germany).

### Oil Red O Staining

Briefly, cells were plated on a 12-well plate to reach 50% confluence for preparation, then the culture medium was removed and cells were washed with (PBS). Cells then were fixed in 4% PFA for 30, washed with 60% isopropyl alcohol for 10 s, and stained with ORO working solution (Sigma) for 15 min. After two times of washing with 60% isopropyl alcohol and PBS, the nuclei were stained with hematoxylin for 2 min. Cells were imaged using a light microscope (Olympus, Tokyo, Japan). All of these procedures were performed at RT.

### Cholesterol Quantification

The quantification of cellular cholesterol was conducted using an Amplex Red cholesterol assay kit (Catalog no. A12216, Invitrogen) according to the manufacturer’s protocol and a previous study [37, 38]. Briefly, cells were seeded on a six-well plate and harvested using a RIPA lysate after overnight growth; then, the cell lysate was centrifuged at 12000 g, 4 °C, for 10 min. The samples were incubated in dark at 37 °C for 1 h after adding an Amplex Red cholesterol-detecting reagent, and then, total cholesterol content was determined using a fluorescence microplate reader at 560-nm excitation, and 590-nm emission wavelengths. Cholesterol content was normalized to protein content as measured by the BCA protein assay kit.

### RT-PCR

Total RNA from cells was extracted using RNAiso Plus Kit (Takara, Japan); reverse transcription was carried out using PrimeScript™ RT Master Mix (Takara, Japan); and qPCR was performed with TB Green® Premix Ex Taq™ (Takara, Japan), all following the manufacturer’s instruction. The mRNA of the target gene was quantified in three independent cell cultures. And the value of threshold cycle (Ct) was acquired by Sequence Detection Software V1.4 (7300 System, Applied Biosystems, USA). The comparative ΔΔCt method was used to analyze ACTIN-normalized expression levels of the target mRNA. Primers of actin were obtained from Sangon, China (B661302-0001). Primer sequences of other genes of interest are shown in Table 1.

**Table 1:** PCR primer sequences of genes involved in cholesterol metabolism

Genes		Primer sequences
SQLE	Forward	AGTTCGCTGCCTTCTCGGATA
	Reverse	GCTCCTGTTAATGTCGTTTCTGA
HMGCR	Forward	ATGCCTTGTGATTGGAGTTG
	Reverse	GTTACGGGGTTTGGTTTATT
SREBP2	Forward	GCAGCAACGGGACCATTCT
	Reverse	CCCCATGACTAAGTCCTTCAACT
ACAT	Forward	TCCACTCCATGCACCACAGTAAA
	Reverse	CGCCTGCCACCATCACATCC
DHCR7	Forward	AGGCTGGATCTCAAGGACAAT
	Reverse	GCCAGACTAGCATGGCCTG
DHCR24	Forward	CGCTGCGAGTCGGAAAGTA
	Reverse	GTCACCTGACCCATAGACACC

### Statistical analysis

Statistical analyses were performed using GraphPad Prism 8.0 (GraphPad Software, USA). To compare statistical differences among each group, one-way ANOVA was used. The definition of statistical significance is when *P < 0.05. All bar graphs are presented as the mean ± SEM.

## Results

### DHCR24 Knockdown and Knock-in in C8D1A Astrocytes was Driven by Lentivirus-Mediated DHCR24-shRNA or DHCR24-cDNA

**Fig. 1:**
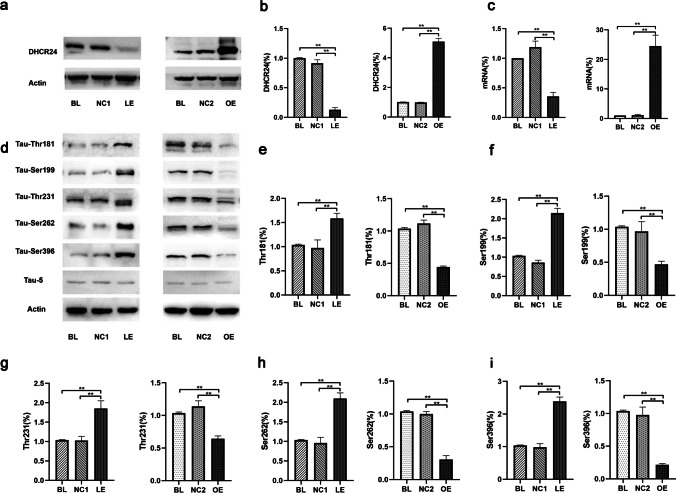
DHCR24 low expression induced tau hyperphosphorylation at specific sites in C8D1A astrocytes**. a, b** Expression of DHCR24 protein was measured by Western blot of cell lysates from DHCR24 low expression (LE) and overexpression (OE) group, compared with blank control (Blank, BL) and vector groups (Negative control, NC1 or NC2). **c** Expression of DHCR24 mRNA was verified by quantitative PCR. The values are shown as the means ± SEM, *n* = 3 per group. **d** Representative images of Western blot analysis of phosphorylated tau. Phosphorylation of tau at Thr181, Ser199, Thr231, Ser262, and Ser396 sites was measured among all groups. **e**, **f, g, h, i** One-way ANOVA analysis of tau phosphorylation level at Thr181, Ser199, Thr231, Ser262 and Ser396 after DHCR24 knockdown or knock-in. The values are shown as the means ± SEM, *n* = 4 per group, One-way ANOVA, *P < 0.05; **P < 0.01; compared with blank control (blank, BL) and vector groups (negative control, NC1 or NC2)

To explore the role of DHCR24 expression on tau phosphorylation in astrocytes, DHCR24 was knocked down by DHCR24-shRNA in C8D1A cell lines. In addition, DHCR24 was overexpressed using a pLVX-Puro lentiviral vector containing DHCR24-cDNA. In DHCR24 lower-expressing C8D1A astrocytes, the DHCR24 mRNA expression level decreased by 65%, compared to blank control (Blank, BL) and vector group (negative control, NC1) cells. And the DHCR24 protein level was significantly reduced (decreased to 12%) (Fig. 1a), suggesting the successful inhibition of DHCR24 expression in this cell line. In contrast, analysis of DHCR24 mRNA revealed an obvious increase (25.5 ± 0.32, *P* < 0.01) in DHCR24-overexpressing cells (Fig. 1c). A significant increase of 5.094-fold in the DHCR24 protein level was found in DHCR24 knock-in cells when compared with blank control (blank, BL) and vector group (negative control, NC2) cells. Altogether, these results suggest DHCR24 knockdown and knock-in C8D1A astrocytes were successfully generated.

### DHCR24 Knockdown Induced Tau Hyperphosphorylation in C8D1A Astrocytes

Tau tauopathy in astrocytes from patients with Alzheimer’s disease or other tauopathies were detected to be phosphorylated at sites of Thr181, Ser199, Ser202, Ser214, Thr231, Ser262, Ser396, and Ser422 [7, 11]. To figure out whether DHCR24 knockdown affects tau phosphorylation in astrocytes, we assessed the tau phosphorylation level at Thr181**,** Ser199**,** Thr231, Ser262, and Ser396 sites, as these sites have been tightly associated with tau pathology in AD [6, 39]. Phosphorylation sites of tau were measured by Western blot analysis. As shown in Fig. 1d, when C8D1A cells were transfected with the lentivirus expressing DHCR24-shRNA (LE), increased phosphorylation of tau at Thr181**,** Ser199**,** Thr231, Ser262, and Ser396 was observed, as compared to the control groups (BL and NC1). Of note, among these sites, the phosphorylation level of Ser396 reached its maximum (2.38-fold). In contrast, Thr181 was the least affected site after DHCR24 knockdown (only 1.59-fold). Moreover, quantitative analysis showed that the phosphorylation level of tau Ser199**,** Thr231, and Ser262 was increased by 2.15 ± 0.12, 1.85 ± 0.20, and 2.01 ± 0.14, respectively, in DHCR24 lower-expressing C8D1A cells. Thus, these findings suggest that DHCR24 downregulation could cause tau hyperphosphorylation in astrocytes. In addition, we further tested whether increasing DHCR24 levels would reverse the above effects. As shown in Fig. 1d, in astrocytes overexpressing DHCR24, we found that the phosphorylation level of tau at Thr181**,** Ser199**,** Thr231, Ser262, and Ser396 were all dramatically decreased, leading to a 55.72%**,** 53.01%**,** 35.72%, 69.26%, and Ser396 78.42% decrease in tau phosphorylation, respectively. The above outcomes suggest that DHCR24 overexpression could suppress tau hyperphosphorylation in C8D1A astrocytes. Furthermore, we observed no alteration in the protein level of total tau among all groups, as recognized by the Tau5 antibody. Altogether, our findings unveil for the first time that DHCR24 is involved in the regulation of astrocytic tau phosphorylation in C8D1A astrocytes.

### DHCR24 Downregulation Leads to Decreased Cellular Free Cholesterol Content in C8D1A Astrocytes

**Fig. 2:**
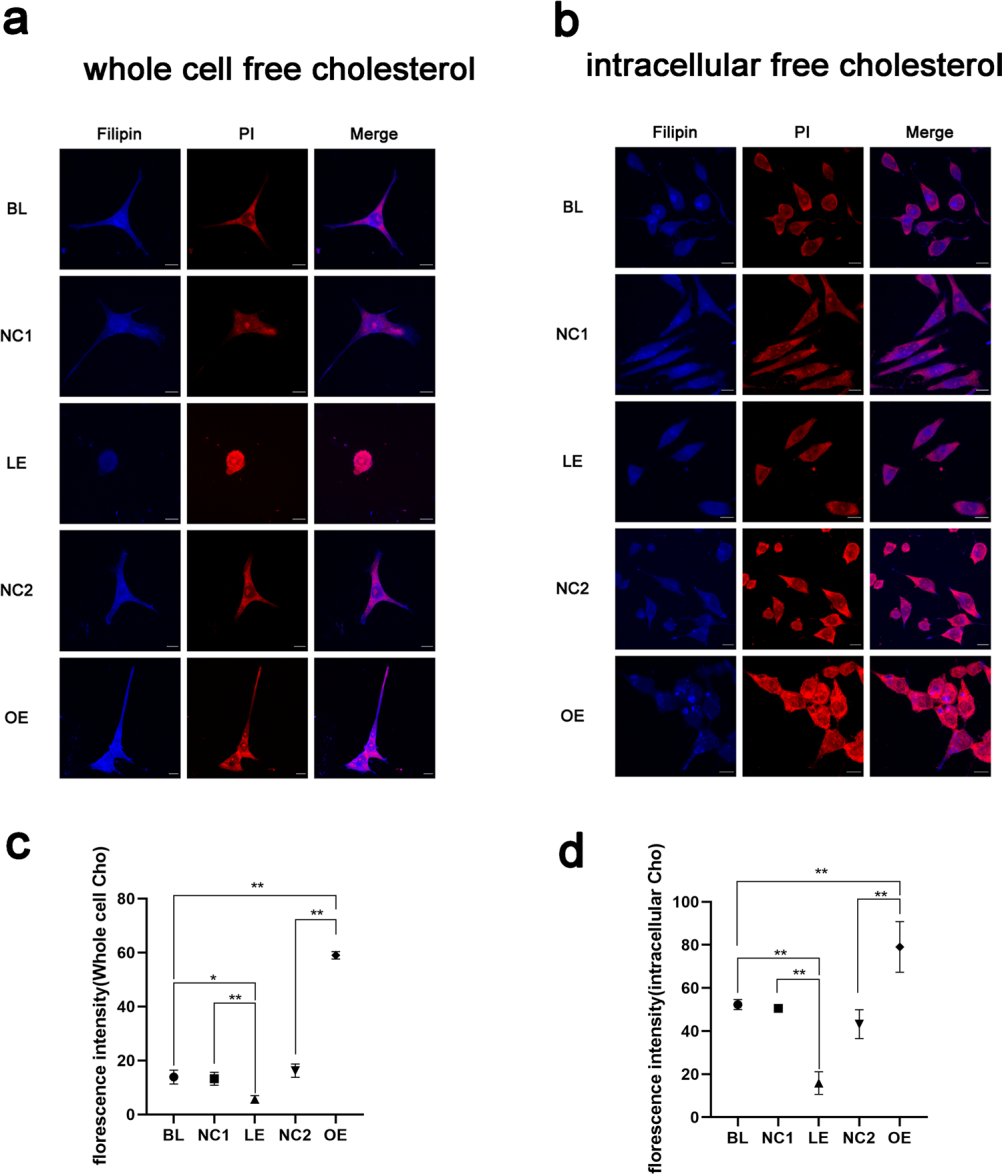
DHCR24 downregulation decreased cellular free cholesterol level in C8D1A astrocytes. **a** Filipin III staining of whole cell free cholesterol. Cells from DHCR24 low expression (LE) and overexpression (OE) group, also the blank control (Blank, BL) and vector groups (Negative control, NC1 or NC2) were stained with filipin III, which could label whole cell free cholesterol, including cholesterol in the plasma membrane. **b** Filipin III staining of intracellular pool of free cholesterol. Before the staining, cells were treated with MβCD for 30 min, which could delete plasma membrane cholesterol to allow the intracellular pool of cholesterol visible. Filipin III (Blue), PI (Red), Magnification: 63 × , Scale bar: 10 µm. The images were captured at random and analysis of filipin fluorescent intensity was shown in (**c**) (whole cell free cholesterol) and (**d**) (intracellular free cholesterol). One-way ANOVA, *P < 0.05; **P < 0.01; compared with blank control (blank, BL) and vector groups (negative control, NC1 or NC2)

Given that DHCR24 is such a crucial molecule regulating cholesterol de novo synthesis, we next assess the change of cellular free cholesterol content by Filipin III staining. Filipin III is a florescent that can specifically label cellular free cholesterol, including cholesterol in the plasma membrane [36]. As shown in Fig. 2a, by assessing the whole cell free cholesterol level using the fluorescent probe Filipin III, we observed that cells infected with lentivirus expressing DHCR24-cDNA exhibited significantly higher levels of fluorescence compared to the control culture (BL and NC2), suggesting that DHCR24 overexpression induced the biosynthesis of cholesterol. Moreover, compared to control cells, the plasma membrane that positively stained with Filipin III was thicker in DHCR24-overexpressing astrocytes, which means enhanced cellular cholesterol synthesis also leads to increased cholesterol content in the plasma membrane. In contrast, much weaker fluorescent signals were detected in the DHCR24-shRNA-infected cells, indicating that DHCR24 deficiency was able to reduce the cellular cholesterol level. In addition, cells were cultured in a medium containing methyl-β-cyclodextrin (MβCD) for 30 min to deplete cholesterol in the plasma membrane before staining, so as to make the intracellular pool of free cholesterol visible [40]. Similarly, after MβCD treatment, fluorescence staining analysis revealed that overexpression of DHCR24 increased the level of intracellular free cholesterol (Fig. 2b). Cells overexpressing DHCR24 exhibited more coarse cytoplasmic granules; while in control cells, cytoplasmic granules were less and tiny. On the contrary, the elevation of intracellular cholesterol was reversed by DHCR24 knockdown. Cytoplasmic cholesterol particles can barely be observed in the lower-expressing DHCR24 cell group. Taken together, the above results indicate that the cholesterol levels of the plasma membrane and intracellular organelles are affected by the change of DHCR24 expression.

### DHCR24 Knockdown Initiated Feedback Regulation of Cholesterol Biosynthesis in C8D1A Astrocytes

**Fig. 3:**
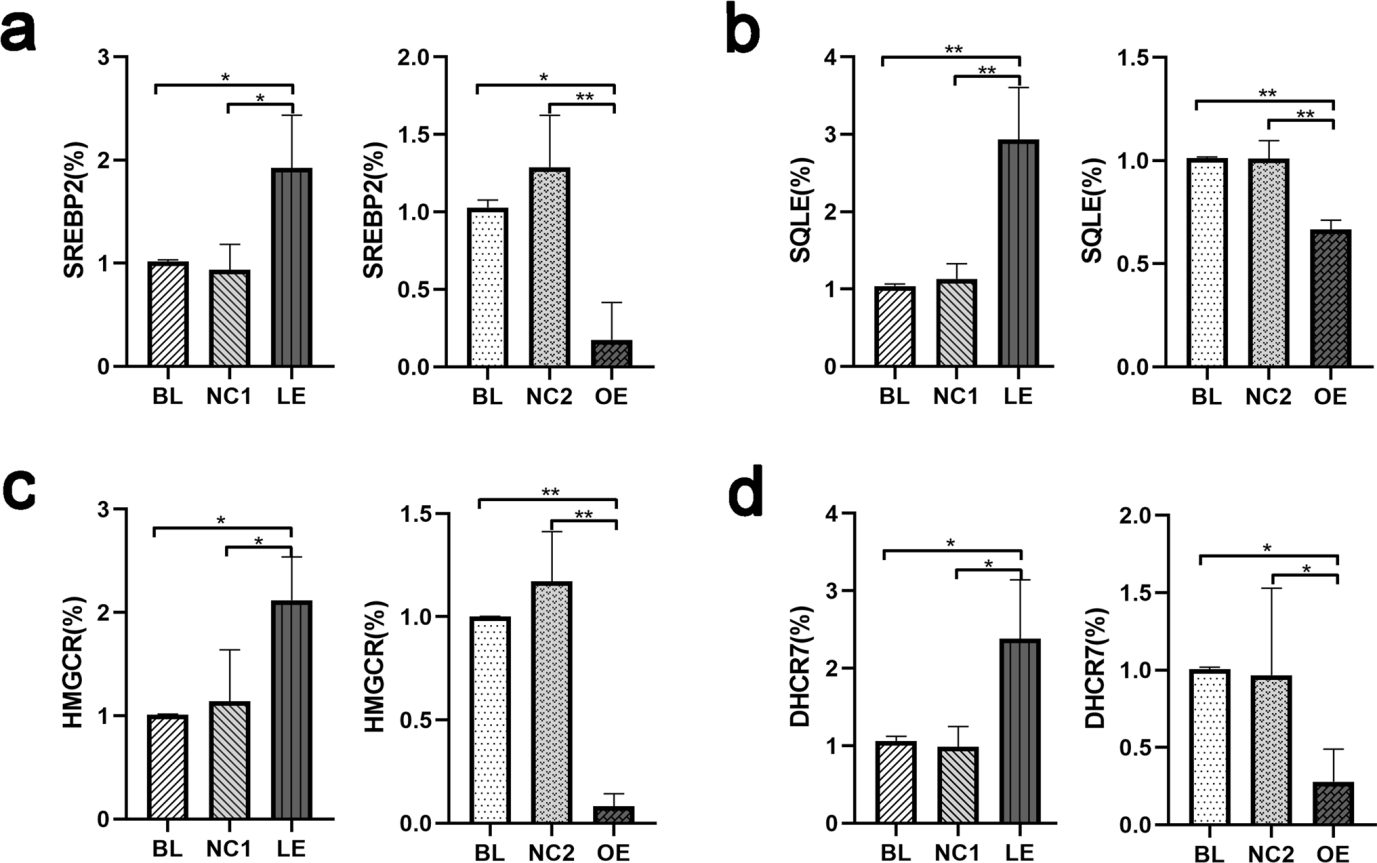
DHCR24 low expression enhanced the transcription of cholesterol key synthetases in C8D1A astrocytes**. a**, **b**, **c**, **d** qPCR analysis of the mRNA level of several key cholesterol synthetases, such as SREBP2, SQLE, HMGCR and DHCR7, in DHCR24 low expression (LE) and overexpression (OE) cells. The values are shown as the means ± SEM, *n* = 3, One-way ANOVA analysis, *P < 0.05; **P < 0.01; compared with control groups (BL, NC1 and NC2)

As twenty-some enzymes are involved in cholesterol synthesis, and previous studies have shown that feedback regulation of these enzymes initiates when cellular cholesterol content is altered [41], we therefore question whether modulating DHCR24 expression acts on the transcription of other enzymes associated with cholesterol metabolism. The mRNA level of 3-hydroxy-3-methylglutaryl coenzyme A reductase (HMGCR), squalene monooxygenase (SQLE), sterol regulatory element-binding protein 2 (SREBP2), and 7-dehydrocholesterol reductase (DHCR7) was measured by semiquantitative RT-PCR after DHCR24 knockdown or knock-in, which are rate-limiting enzymes or crucial regulator of cholesterol biosynthesis. As shown in Fig. 3, in DHCR24-silencing C8D1A astrocytes, HMGCR, SQLE, SREBP2, and DHCR7 mRNA expression levels were significantly increased by 2.1-, 2.9-, 1.9-, and 2.3-fold, respectively, compared to vector cells. Conversely, DHCR24 overexpression markedly decreased their mRNA levels compared with control cells. Thus, these findings suggest that DHCR24 downregulation seems to promote the transcription of other cholesterol synthetases such as SREBP2, HMGCR, SQLE, and DHCR7, suggesting a secondary and compensatory response due to cholesterol deficiency by DHCR24 knockdown.

### DHCR24 Downregulation Inhibited Intracellular Cholesterol Esterification in C8D1A Astrocyte

**Fig. 4:**
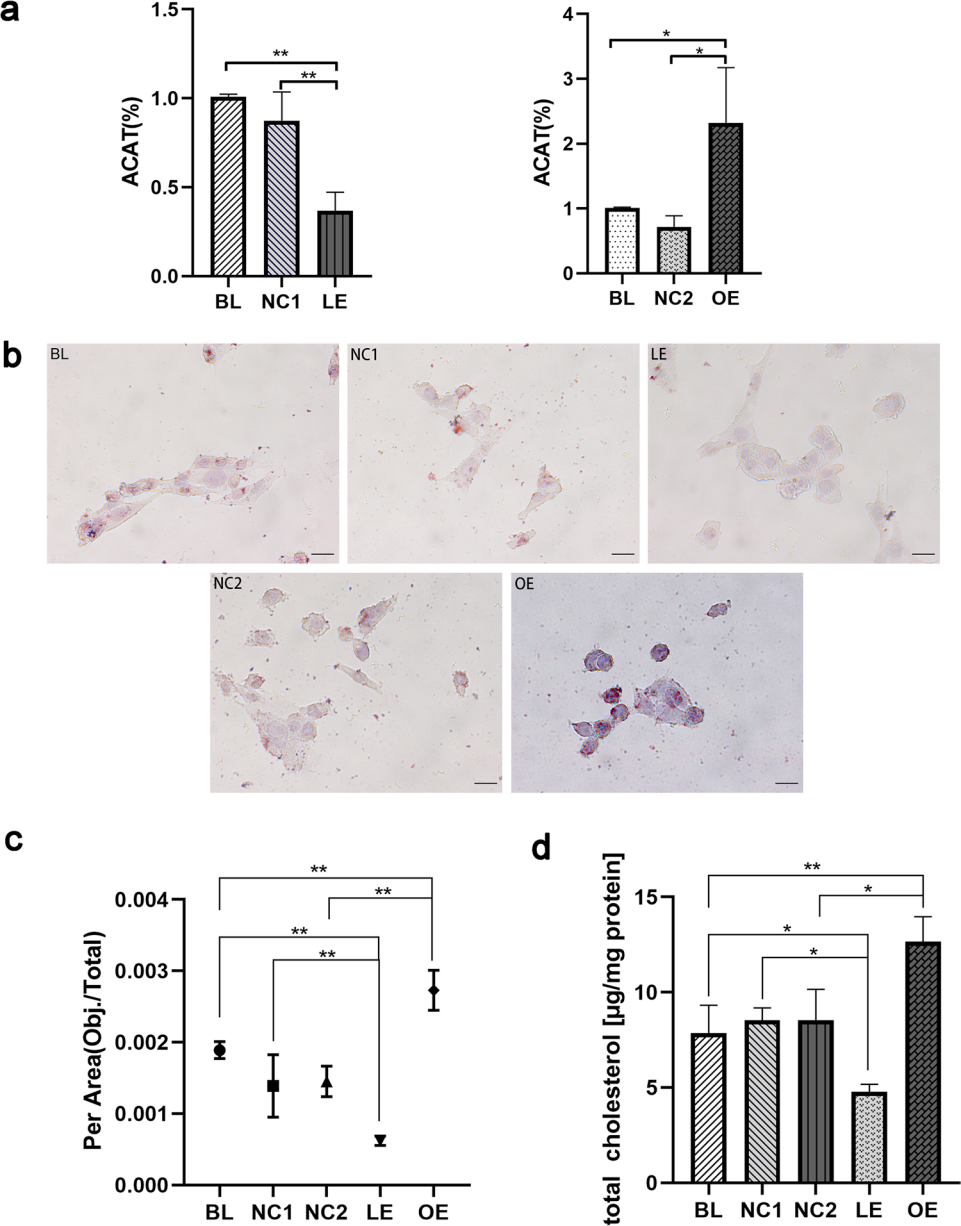
DHCR24 downregulation reduced cholesterol esterification and total cholesterol content in C8D1A astrocytes. **a** Transcription level of ACAT was measured by qPCR analysis. The values are shown as the means ± SEM, n = 3 per group. **b** Representative images of Oil Red O staining of lipid droplets of astrocytes from blank control group (Blank, BL) and vector groups (Negative control, NC1 or NC2), DHCR24 low expression (LE) and overexpression (OE) groups. Magnification: 60 × , Scale bar: 20 µm. The images were captured at random, and the average number of lipid droplets from each group was analyzed using Image Pro Plus 6.0 version. One-way ANOVA analysis of the results was shown at (**c**). **d** Quantification of total cholesterol content. One-way ANOVA analysis, *P < 0.05; **P < 0.01; compared with control groups (BL, NC1 and NC2)

We additionally assessed the mRNA level of acyl coenzyme A:cholesterol acyltransferase (ACAT), which plays an important role in maintaining cellular cholesterol homeostasis. Abundant cellular cholesterol serves as an ACAT activator, and then, this enzyme esterifies free cholesterol to cholesterol esters which can be stored in lipid droplets [41, 42]. The data showed that knockdown of DHCR24 caused a significant decrease (63% lower) in the ACAT mRNA level compared with control C8D1A cells (Fig. 4a), suggesting that DHCR24 knockdown inhibited the transformation of cholesterol to cholesterol esters. On the contrary, DHCR24 overexpression obviously increased the ACAT mRNA level (2.3-fold) compared with the vector group in C8D1A astrocytes, therefore promoted the conversion of cholesterol into its storage form. So, it is apparent that DHCR24 expression acts on both the biosynthesis and esterification of cholesterol, which makes it a noticeable regulator of cholesterol homeostasis. Collectively, once the expression of DHCR24 is altered, negative feedback regulation of cholesterol synthesis and esterification is initiated.

To further explore the change of cholesterol esterification after DHCR24 knockdown, we performed Oil Red O staining to analyze the change in the intracellular cholesterol ester content. Oil Red O is a fat-soluble dye that specifically stains triglycerides and cholesteryl oleate but no other lipids [43]. Hence, this method is usually applied in the visualization of lipid droplets. Previous studies have shown that the number of cellular lipid droplets increases with cholesterol ester accumulation [44, 45]. As shown in Fig. 4b, Oil Red O staining revealed that a significantly increased number of lipid droplets could be observed in overexpressing DHCR24 C8D1A cells, as compared to control cells, suggesting enhanced esterification of cholesterol with DHCR24 upregulation. Reversely, almost no lipid droplets could be stained in cells infected with DHCR24-shRNA, which indicates the deficiency of cholesterol esters compared to the control cells. Consistent with the changes in ACAT transcription in a previous experiment (Fig. 4a), DHCR24 overexpression could enhance ACAT expression and then promotes obsessive cellular free cholesterol to transform into cholesterol esters; while DHCR24 knockdown could inhibit ACAT expression and cholesterol esterification. Taken together, DHCR24 knockdown induced the decrease in cellular cholesterol ester content due to the decrease in cellular cholesterol level in C8D1A astrocyte.

### Silencing DHCR24 Led to Decreased Cellular Cholesterol Content

We at a previous experiment have observed that DHCR24 downregulation could lead to decreased cellular free cholesterol levels and reduced cholesterol esters, as shown by Filipin staining and Oil Red O staining. And the altered transcription level of enzymes involved in cholesterol metabolism has also suggested the disruption of cellular cholesterol homeostasis. To further verified that cholesterol homeostasis was disturbed after the change of DHCR24 expression, we next applied Amplex Red cholesterol quantification kit to assess total cholesterol content. As shown in Fig. 4d, the experiment revealed that total cholesterol was decreased with DHCR24 downregulation while DHCR24 upregulation significantly increased total cholesterol level, compared with control groups (BL: 7.861 ± 1.461 vs. NC1: 8.535 ± 0.64 vs. LE: 4.784 ± 0.380 vs. NC2: 8.539 ± 1.613 vs. OE: 12.650 ± 1.314). Therefore, alteration of DHCR24 expression could remarkedly affected cellular cholesterol content by interrupting cholesterol biosynthesis.

### DHCR24 Knockdown Disrupted Caveolae and Activated the MAPK/ERK Signaling Pathway in C8D1A Astrocytes

**Fig. 5:**
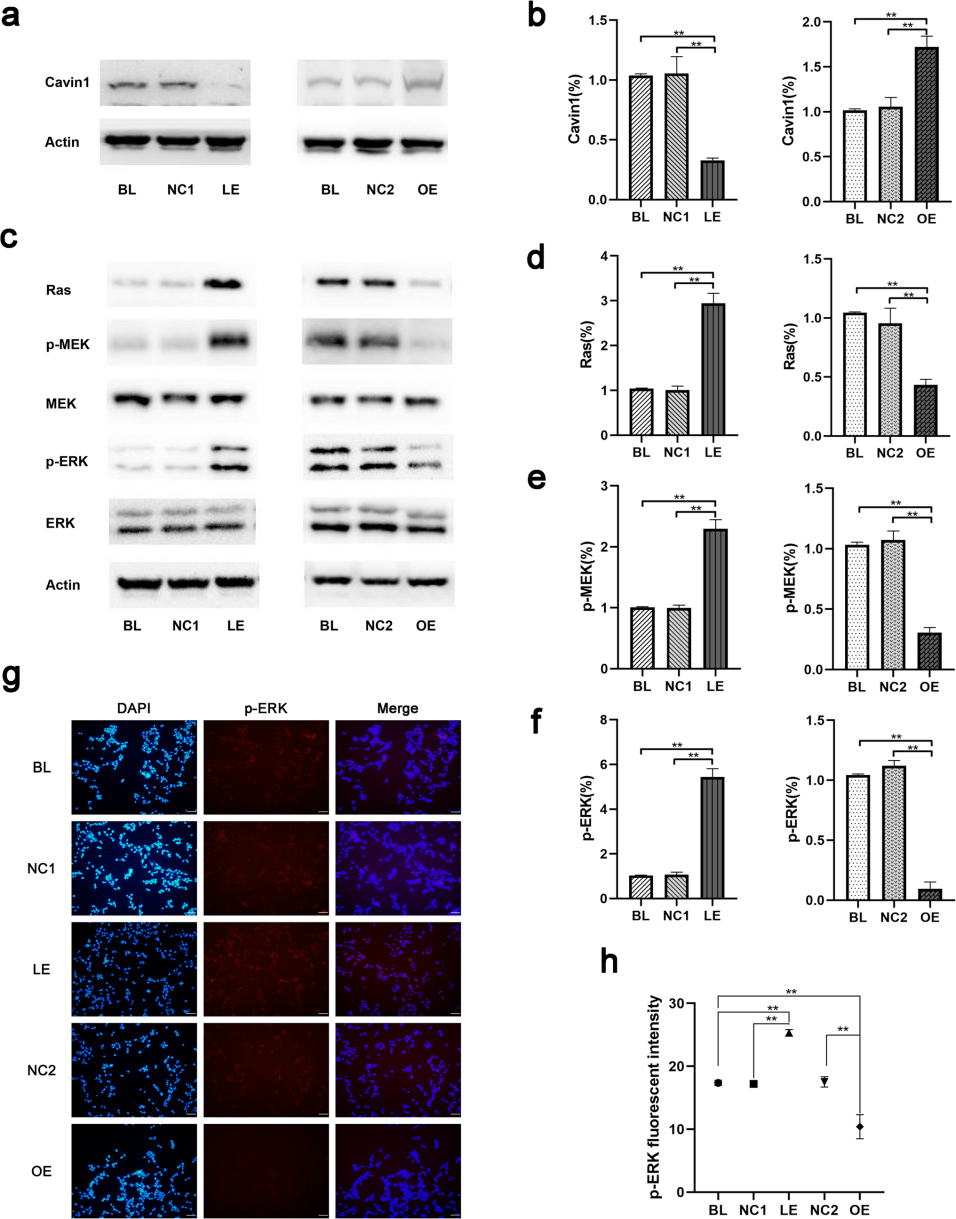
DHCR24 downregulation disrupted caveolae and activated Ras/MEK/ERK signaling pathway in C8D1A astrocytes. **a** Expression of cavin1 was measured by Western blot of cell lysates from DHCR24 DHCR24 low expression (LE) and overexpression (OE) groups, *n* = 4 per group. One-way ANOVA analysis of cavin1 expression level was shown in (**b**). The values are shown as the means ± SEM, *n* = 4 for each group. **c** Ras, total MEK (MEK), phosphorylated MEK (p-MEK), total ERK1/2 (ERK), and phosphorylated ERK1/2 (p-ERK) protein levels were measured by Western blot. One-way ANOVA analysis of their expression among all groups is shown in (**d**, **e**, **f**), *n* = 4 per group. **g** Representative images of immunofluorescence staining of p-ERK (Red); Magnification: 20 × , Scale bar: 50 µm. The images were captured at random and analysis of p-ERK fluorescent intensity was shown in (**h**). One-way ANOVA, *P < 0.05; **P < 0.01; compared with blank control (blank, BL) and vector groups (negative control, NC1 or NC2)

Given that cholesterol is a major component of caveolae, and the content of plasma membrane cholesterol was affected when DHCR24 expression was altered (Fig. 2a), we postulated DHCR24 knockdown could disrupt caveolae formation. It has been identified that cavin1 is one of the most important lipid-raft proteins in cholesterol-dependent caveolae formation, ablation of its expression leads to the disruption of caveolae [32], and the existence of cavin1 is essential for other caveolae-associated protein to maintain caveolae formation [46]. A series of studies previously identified that DHCR24 downregulation led to decreased expression of structural protein of caveolae, such as caveolin1[19, 28, 44], whereas the relationship between DHCR24 expression and cavin1 remains unknown. For this purpose, cavin1 protein level was measured by Western blot analysis (Fig. 5a). Our data showed that expression of cavin1 was enhanced in cells overexpressing DHCR24. However, depletion of DHCR24 in C8D1A astrocytes caused decreased cavin1 protein level, suggesting caveolae formation might be disrupted. Based on the previous experimental data, our findings together imply that decreased cholesterol content in the plasma membrane by DHCR24 downregulation might lead to the disruption of caveolae.

It has been suggested that activation of MAPK/ERK promotes tau phosphorylation, and this signaling pathway was activated in AD pathology and in astrocytes bearing phosphorylated tau [47–49]. Therefore, we investigated whether Ras/MEK/ERK signaling is involved in DHCR24-deficiency-mediated tau phosphorylation in astrocytes. As shown in Fig. 5c, we found greater expression of Ras in lower-expressing DHCR24 cells than in the control cells, while Ras protein level was dramatically reduced in DHCR24 knock-in cells. Moreover, we then measured protein level of phosphorylated MEK (p-MEK) and ERK1/2 (p-ERK), which are downstream molecules activated by Ras. Unsurprisingly, our outcomes demonstrated that phosphorylation of both MEK and ERK were notably increased in DHCR24-silencing cells, suggesting the activation of MEK/ERK signaling pathway. In contrast, the data showed that knock-in of DHCR24 caused significant decreases in p-MEK and p-ERK protein levels compared with control cells, which indicates that DHCR24 overexpression could prevent the overactivation of MEK and ERK. Total protein level of MEK and ERK remained unchanged among all groups. In addition, to further assess the activation of MAPK/ERK signaling pathway, immunofluorescence staining of p-ERK was performed. As shown in Fig. 5g, we observed that cells infected with lentivirus expressing DHCR24-shRNA exhibited significantly higher levels of fluorescence compared to the control culture (BL and NC1), suggesting that DHCR24 deficiency induced the phosphorylation of ERK. Conversely, much weaker fluorescent signals were detected in the DHCR24 knock-in cells, indicating that DHCR24 upregulation was able to reduce ERK activation. Taken together, DHCR24 knockdown activates the caveolae Ras/MEK/ERK signaling pathway in C8D1A astrocytes.

### Inhibition of MEK/ERK Signaling Reduced Tau Hyperphosphorylation Induced by DHCR24 Knockdown in C8D1A Astrocytes

**Fig. 6:**
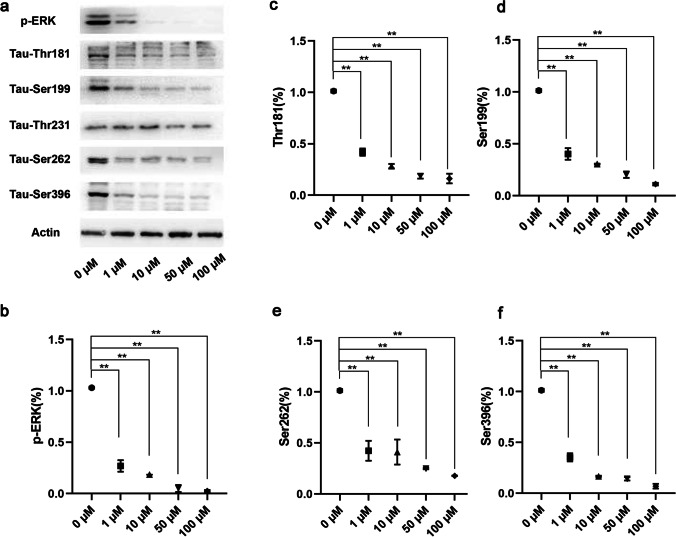
Inhibition of MEK/ERK signaling reduced tau hyperphosphorylation in DHCR24 knockdown C8D1A astrocytes. **a** Western blot analysis was applied to assess the protein level of phosphorylated ERK (p-ERK) and phosphorylated tau. DHCR24 knockdown cells were cultured with MEK inhibitor U0126. After 24 h, the phosphorylation level of tau at Thr181, Ser199, Ser262, Thr231, and Ser396 sites were verified. **b**, **c**, **d**, **e**, **f** One-way ANOVA analysis of Western blot results of p-ERK and phosphorylated tau. The values are shown as the means ± SEM, *n* = 4 for each group. *P < 0.05; **P < 0.01; compared with blank control (0 µM)

To determine whether activation of Ras/MEK/ERK signaling pathway is involved in tau hyperphosphorylation after DHCR24 knockdown in astrocytes, we applied a known MEK inhibitor, U0126, in the culture medium of DHCR24 lower-expressing cells. U0126 is capable of preventing the activation of MEK1 and MEK2, selectively, thus the subsequent inactivation of p-ERK [50]. DHCR24 knockdown cells were incubated with gradient concentrations of 0 µM, 1 µM, 10 µM, 50 µM, 100 µM of the inhibitor for 24 h. Afterward, cells were extracted for protein and phosphorylation sites of tau were mapped by Western blot analysis. We at first measured the protein level of p-ERK to determine the efficiency of ERK inhibition. As seen in Fig. 6a, U0126 partially abolished the phosphorylation of ERK at 1 µM, and when it was in higher concentration (10–100 µM), p-ERK can barely be detected, suggesting the successful inhibition of ERK activation at higher concentrations of U0126. Furthermore, we next assessed the level of phosphorylated tau after p-ERK inhibition. As shown in Fig. 6a, when DHCR24 knockdown cells were incubated with U0126, decreased phosphorylation of tau at Thr181**,** Ser199**,** Ser262 and Ser396 was observed, as compared to the control culture. Among these sites, Ser396 was the most affected one because its phosphorylation level reached its minimum, suggesting that MEK/ERK signaling mainly regulates phosphorylation of Ser396 site in DHCR24 knockdown astrocytes. Of note, the phosphorylation of tau at these sites were not completely inhibited even at the highest concentration (100 µM) of U0126 when p-ERK can barely be detected, which implies that MEK/ERK signaling partially regulates tau phosphorylation in C8D1A astrocytes. In other words, multiple kinases or signaling pathway could together contributes to tau phosphorylation, which is in line with previous research [33, 39, 51, 52]. In addition, we also surprisingly found no alteration in Thr231 protein level after U0126 application, suggesting that activated MEK/ERK signaling has no effects on Thr231 phosphorylation in DHCR24 knockdown astrocytes. Collectively, our results identified that DHCR24 downregulation in astrocytes could activate MEK/ERK pathway to enhance tau phosphorylation at Thr181, Ser199, Ser262 and Ser396, but not Thr231.

## Discussion

In the present study, we found that silencing DHCR24 could lead to tau hyperphosphorylation at Thr181, Ser199, Thr231, Ser262 and Ser396 sites, while over-expressing DHCR24 could reverse the effects of tau phosphorylation at these sites by DHCR24 knockdown in astrocyte C8-D1A Cells. Tau-bearing astrocytes have been observed in the aged population, AD mouse model and AD patients, and overexpression of tau in astrocytes caused exhibition of abnormally phosphorylated and pathological conformation of tau [6, 7, 34, 53, 54]. Besides, it was found that pathological neuronal tau cannot be seeded to astrocytes, suggesting that tau hyperphosphorylation could be, at least partly, produced in astrocytes itself [14, 15]. For decades, plenty of evidence have suggested that tau hyperphosphorylation at these sites were identified in astrocytic tau pathological deposition [6, 11, 55]. Similarly, previous data has demonstrated that these sites have also been identified in neuronal tau pre-tangles and tangles, which contribute to the tau pathology in AD and other tauopathies [56–58]. And tau hyperphosphorylation and accumulation in astrocytes has been suggested to be a degenerative instead of reactive process, for astrocytic tau inclusion was independent of reactive astrocytes [59]. Till now, different ways of tau propagation have been identified, one of which is induced by secretion of tau-containing exosomes [60]. It has been found that astrocytes-derived exosomes contain pathologically phosphorylated tau, and astrocytes could release phosphorylated tau with or without pathological stimulation [4, 61, 62]. Moreover, injection of astrocytic pathological tau leads to remarkable transmission of glia tau inclusion in non-transgenic mice [14]. Additionally, astrocytic tau accumulation was found to cause cellular disfunction, such as mitochondria dysfunction, reduced neurogenesis, mild blood- brain barrier disruption, focal neuron degeneration and instability and collapse of cytoskeleton, tau-bearing astrocytes could eventually lead to language and spatial dysfunction, as well as memory deficits [54, 63]. Based on previous data and our finding, astrocytic tau pathology could bring about a pathological change in the astrocyte itself. Taken together, our findings support a new concept that astrocytes itself can produce hyperphosphorylated tau, which might be involved in astrocytic tau pathology.

In previous study, we found that DHCR24 knockdown could lead to hyperphosphorylation of tau in neuronal SHSY-5Y cells, which is tightly related to cell cholesterol deficiency [19, 31]. However, in their studies, Filipin III staining is the only method used to reflect cellular cholesterol levels. While in the present study, we used additional Oil Red O staining, qPCR analysis of crucial enzymes that regulate cholesterol metabolism and quantification of total cholesterol level to assess dysregulated cholesterol homeostasis. In the present experiment, we found that silencing DHCR24 could also markedly reduce whole cellular free cholesterol levels, including cholesterol in the plasma membrane and intracellular cholesterol in astrocytes, as revealed by Filipin III staining. Furthermore, we did find that the transcription of key cholesterol synthetases, including SREBP2, HMGCR, SQLE, and DHCR7, was significantly increased after DHCR24 knockdown, and markedly decreased after DHCR24 knock-in, suggesting that the change of DHCR24 expression alters cholesterol content in astrocytes. Previous research suggests that when endoplasmic reticulum (ER) cholesterol is depleted, the expression of rate-limiting enzymes in cholesterol synthesis will be enhanced [41, 64, 65]. Conversely, when cellular cholesterol content exceeds 5% of total lipids in ER, the negative feedback regulation of cholesterol synthesis will be initiated [66]. So, the above data further demonstrated that DHCR24 knockdown could lead to a deficiency of cellular cholesterol. In addition, by Oil Red O staining analysis, we also found that DHCR24 knockdown induced the decrease in intracellular cholesterol esters, and DHCR24 knock-in increased the level of intracellular cholesterol esters in C8-D1A astrocytes. Meanwhile, in line with Oil Red O staining, the mRNA level of ACAT was decreased in DHCR24-silencing cells, whereas the transcription of this gene was upregulated in DHCR24-overexpressing C8D1A astrocytes. The above finding supports a deficiency of cellular cholesterol by DHCR24 knockdown leads to the decrease in cholesterol esterification, which is consistent with previous studies [42, 44, 45, 67]. We additionally applied a cholesterol quantification kit to further verified the alteration of total cholesterol content after changes in DHCR24 expression. Consistent with Filipin staining, Oil Red O staining, and qPCR analysis, the quantification of total cellular cholesterol confirmed that DHCR24 downregulation did reduce the cholesterol level significantly. Collectively, our outcomes combined together confirmed that silencing DHCR24 could lead to inhibition of cholesterol synthesis and remarkably decreased cellular cholesterol level, coupled with feedback enhancement of other cholesterol synthetases activity and decrease in cholesterol esterification, resulting in dysregulation of cholesterol homeostasis in astrocytes. Thus, we firstly established an in vitro astrocytic glial cell model of cellular cholesterol deficiency by lentivirus-mediated knockdown of DHCR24.

Cholesterol is the major component of the cellular membrane and has been indicated to affect the construction and function of caveolae [68–70]. In our experiment, the lower expression of DHCR24 obviously lowered the cholesterol level in the plasma membrane. Here, we showed that DHCR24 knockdown also reduced the protein level of cavin1 in the plasma membrane. Cavin1 is an indispensable protein that contributes to the formation of caveolae [46, 71, 72]. Additionally, a series of research reveal that the absence of cavin1 causes the loss of the caveolae [32, 73], and the existence of this protein is essential for the sequestration of other caveolae-associated proteins into immobile caveolae to maintain its construction and function, such as caveolin1 [46]. In a word, the decrease in cavin1 after DHCR24 downregulation could theoretically impair the construction of caveolae. Taken together, our findings suggest that DHCR24 knockdown might disrupt the formation of caveolae via the decrease in plasma membrane cholesterol.

In the present study, the overactivation of the Ras/MEK/ERK signaling pathway was observed in DHCR24 knockdown astrocytes, while DHCR24 knock-in reduced the activation of this MAPK/ERK signaling. Caveolae have long been regarded as a vital platform for signal transduction and various signaling molecules locate in this construction. Ras is implied to locate in caveolae and interact with caveolae-associated protein [74–76]. The deficiency of cavin1 could cause activation of Ras in caveolae, by regulating Ras nanoclustering and plasma membrane organization [32]. Hence, the activation of Ras/MEK/ERK signaling in DHCR24 lower-expressing C8D1A astrocytes may attribute to the disruption of caveolae. In addition, the Ras/MEK/ERK pathway has been identified to be activated in AD pathology, and both phosphorylated ERK1 and ERK2 are implied to be associated with tau phosphorylation in neurons and glia cells [47, 48, 51]. In our experiment, we did find that DHCR24 knockdown C8D1A astrocytes exhibited both the activation of the Ras/MEK/ERK pathway and synchronously increased the phosphorylation level of tau. On the contrary, DHCR24 knock-in astrocytes displayed reduced activation of this signaling and decreased tau phosphorylation. In order to reveal whether tau phosphorylation by DHCR24 knockdown is caused by overactivation of Ras/MEK/ERK signaling, we applied the MEK inhibitor U0126 in the culture medium of DHCR24-downregulated cells. We identified that the inhibition of MAPK/ERK signaling reduced the phosphorylation of tau at Thr181, Ser199, Ser262, and Ser396 sites, suggesting that Ras/MEK/ERK signaling, at least partially, contributes to tau hyperphosphorylation in DHCR24-silencing cells. Intriguingly, among those sites of tau phosphorylated by MEK/ERK signaling, Ser396 was the most affected one, which is consistent with a previous study [77]. In addition, although the tau Thr231 site was also hyperphosphorylated after DHCR24 knockdown, the inhibition of MAPK/ERK signaling has no effect on its phosphorylation. The possible explanation is that phosphorylation of tau Thr231 is regulated by other protein kinases or phosphatases, as shown in previous studies [19, 31, 78]. Above all, our findings suggest that DHCR24 knockdown leads to phosphorylation of tau at Thr181, Ser199, Ser262, and Ser396 sites by overactivation of caveolae-dependent Ras/MEK/ERK signaling in C8D1A astrocytes. Multiple kinases or phosphatases are involved in regulating neuronal tau phosphorylation. Previous studies clarified that activation of GSK3β or inactivation of PP2A could contribute to tau hyperphosphorylation in neuronal cells by DHCR24 knockdown [19, 31]. Here, we for the first time proved that DHCR24 downregulation stimulates tau hyperphosphorylation in astrocytes via activation of ERK.

Interestingly, a growing body of evidence reveals that the expression of major genes involved in cholesterol synthesis in the brain was obviously downregulated, including DHCR24, in aged mice, diabetic mice, familial Alzheimer’s disease (FAD) mice, and AD patients, which suggest the decrease in de novo cholesterol synthesis and brain cholesterol loss [20–25]. Furthermore, it has been found that a number of genes involved in cholesterol synthesis are downregulated in aging astrocytes and astrocytes isolated from AD mice, including DHCR24 [25, 27]. In the adult brain, cholesterol is mainly synthesized by astrocytes, and to a lesser extent, neurons [79]. Neurons have a high demand for cholesterol to develop and maintain membrane-rich structures, like axons, dendrites, and synapses, and myelin integrity. However, neurons cannot produce cholesterol efficiently, so they depend on an external supply of cholesterol synthesized by astrocytes [80]. Besides, neurons that are lack of cholesterol would exhibit neurodegenerative changes [81–83]. Obviously, DHCR24 activity in astrocytic glial cells may play a key role in astrocyte-neuron cross talk, particularly in the control of cholesterol synthesis to maintain cholesterol homeostasis in neurons and to maintain the proper function of the brain. Based on our present study, under some pathological conditions, we propose that a decrease in cholesterol biosynthesis in astrocytes by the downregulation of DHCR24 may lead to perturbation of cholesterol efflux in astrocytes and altered astrocyte–neuron cholesterol cross talk, which in turn may affect neuron function. Therefore, cholesterol decrease in astrocytes by DHCR24 knockdown could not only potentially mediate the astrocytic tau pathology, and but also influence neuronal activity.

## Conclusion

**Fig. 7:**
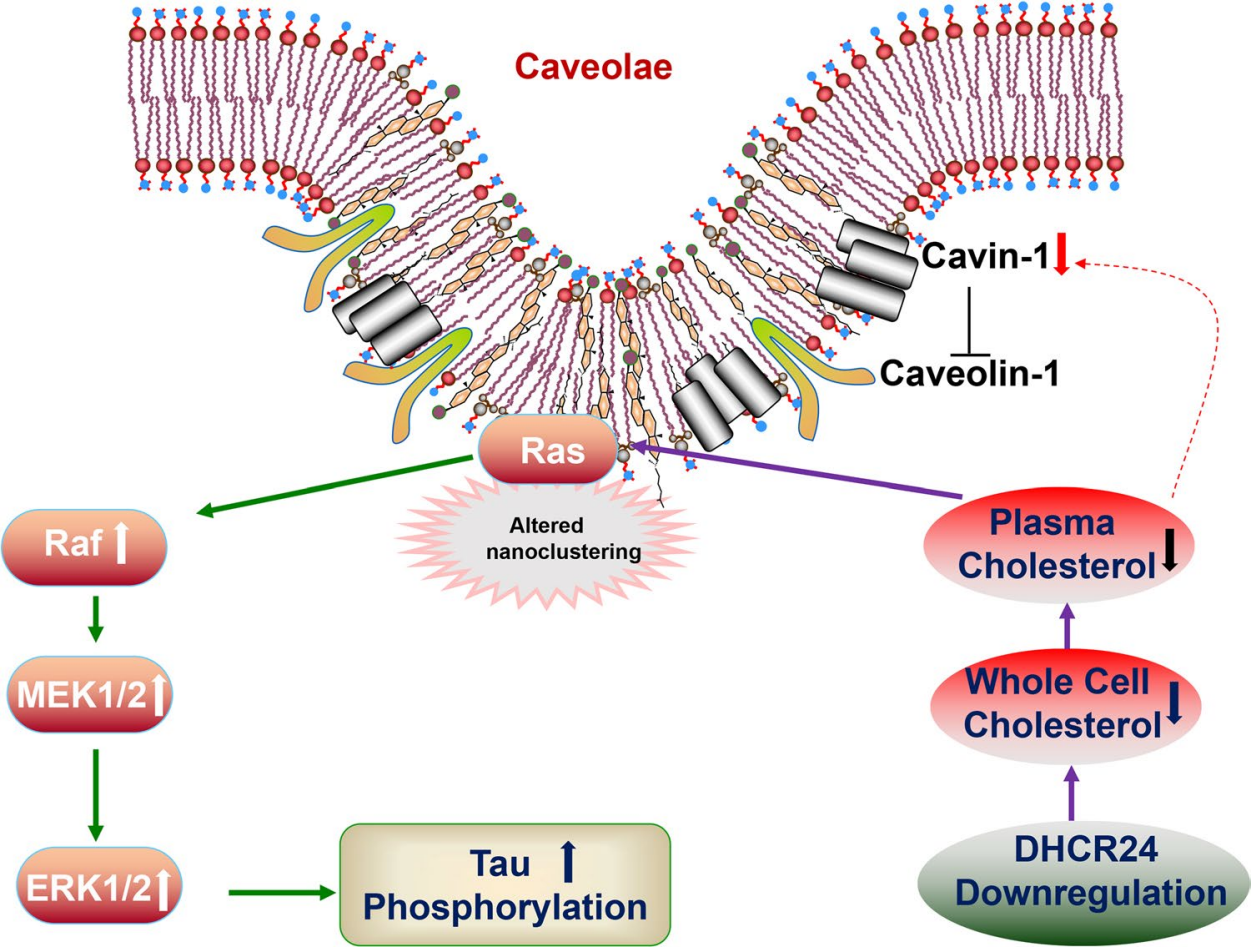
Decreased cellular cholesterol by DHCR24 knockdown contributes to astrocytic tau pathology in C8D1A cells. DHCR24 knockdown in C8D1a astrocytes leads to inhibition of cholesterol synthesis and deficiency of cholesterol in the plasma membrane. Meanwhile, reduced membrane cholesterol decreases the cavin-1 expression level, resulting in the disruption of caveolae. Moreover, the impairment of caveolae construction leads to the altered nanoclustering of Ras that locates in this platform and causes its overactivation, then the MAPK/ERK signaling pathway is activated. Eventually, activated ERK1/2 promotes tau hyperphosphorylation

In conclusion, in astrocytic C8D1A cells, we for the first time established an in vitro astrocytic cell model of cellular cholesterol deficiency by DHCR24 knockdown. We found that DHCR24 knockdown induced the decrease in plasma membrane cholesterol and disruption of lipid raft/caveolae, resulting in activation of lipid raft-dependent Ras/MEK/ERK signaling pathway (Fig. 7). Besides, silencing DHCR24 could markedly promote hyperphosphorylation of tau at some specific sites by activation of Ras/MEK/ERK. Therefore, our findings support that DHCR24 knockdown can lead to tau hyperphosphorylation in the astrocyte itself by activating membrane lipid raft-dependent Ras/ERK/ERK signaling, which might contribute to the pathogenesis of AD and other degenerative tauopathies.

## Data Availability

All data supporting the conclusions of this article are available from the corresponding author on reasonable request.

## Abbreviations

DHCR24: 3β-Hydroxysterol-Δ24 reductase
HMGCR: 3-Hydroxy-3-methylglutaryl coenzyme A reductase
SQLE: Squalene monooxygenase
SREBP2: Sterol regulatory element-binding protein 2
DHCR7: 7-Dehydrocholesterol reductase
ACAT: Acyl coenzyme A:cholesterol acyltransferase
MAPK: Mitogen-activated protein kinase
Ras: A GTPase
MEK: Mitogen-activated protein kinase
ERK: Extracellular signal-regulated protein kinase
shRNA: Short-hairpin RNA
AKT: A serine/threonine-specific protein kinase
PI3K: Phosphoinositide 3-kinase
GSK3β: Glycogen synthase kinase 3 beta
ER: Endoplasmic reticulum
MβCD: Methyl-β-cyclodextrin
PP2A: Protein phosphatase 2A
JNK: C-Jun N-terminal kinase

## References

[1] Couchie D, Charrière-Bertrand C, Nunez J (1988). Expression of the mRNA for tau proteins during brain development and in cultured neurons and astroglial cells. J Neurochem.

[2] Müller R, Heinrich M, Heck S, Blohm D, Richter-Landsberg C (1997). Expression of microtubule-associated proteins MAP2 and tau in cultured rat brain oligodendrocytes. Cell Tissue Res.

[3] Fathi E, Katouli FH, Riazi GH, Shasaltaneh MD, Parandavar E, Bayati S, Afrasiabi A, Nazari R (2017). The effects of alpha boswellic acid on reelin expression and tau phosphorylation in human astrocytes. Neuromolecular Med.

[4] Chiarini A, Armato U, Gardenal E, Gui L, Dal Prà I (2017). Amyloid β-exposed human astrocytes overproduce phospho-tau and overrelease it within exosomes, effects suppressed by calcilytic NPS 2143-further implications for Alzheimer’s therapy. Front Neurosci.

[5] Zhang Y, Chen K, Sloan SA, Bennett ML, Scholze AR, O’Keeffe S, Phatnani HP, Guarnieri P (2014). An RNA-sequencing transcriptome and splicing database of glia, neurons, and vascular cells of the cerebral cortex. J Neurosci.

[6] López-González I, Carmona M, Blanco R, Luna-Muñoz J, Martínez-Mandonado A, Mena R, Ferrer I (2013). Characterization of thorn-shaped astrocytes in white matter of temporal lobe in Alzheimer’s disease brains. Brain Pathol.

[7] García-Matas S, Gutierrez-Cuesta J, Coto-Montes A, Rubio-Acero R, Díez-Vives C, Camins A, Pallàs M, Sanfeliu C (2008). Dysfunction of astrocytes in senescence-accelerated mice SAMP8 reduces their neuroprotective capacity. Aging Cell.

[8] Nakano I, Iwatsubo T, Otsuka N, Kamei M, Matsumura K, Mannen T (1992). Paired helical filaments in astrocytes: electron microscopy and immunohistochemistry in a case of atypical Alzheimer’s disease. Acta Neuropathol.

[9] Ikeda K, Haga C, Akiyama H, Kase K, Iritani S (1992). Coexistence of paired helical filaments and glial filaments in astrocytic processes within ghost tangles. Neurosci Lett.

[10] Ikeda K, Akiyama H, Arai T, Nishimura T (1998). Glial tau pathology in neurodegenerative diseases: their nature and comparison with neuronal tangles. Neurobiol Aging.

[11] Ferrer I, López-González I, Carmona M, Arregui L, Dalfó E, Torrejón-Escribano B, Diehl R, Kovacs GG (2014). Glial and neuronal tau pathology in tauopathies: characterization of disease-specific phenotypes and tau pathology progression. J Neuropathol Exp Neurol.

[12] Ferrer I, Barrachina M, Tolnay M, Rey MJ, Vidal N, Carmona M, Blanco R, Puig B (2003). Phosphorylated protein kinases associated with neuronal and glial tau deposits in argyrophilic grain disease. Brain Pathol.

[13] Kovacs GG (2020). Astroglia and tau: new perspectives. Front Aging Neurosci.

[14] Narasimhan S, Guo JL, Changolkar L, Stieber A, McBride JD, Silva LV, He Z, Zhang B (2017). Pathological tau strains from human brains recapitulate the diversity of tauopathies in nontransgenic mouse brain. J Neurosci.

[15] Wang Y, Balaji V, Kaniyappan S, Krüger L, Irsen S, Tepper K, Chandupatla R, Maetzler W (2017). The release and trans-synaptic transmission of tau via exosomes. Mol Neurodegener.

[16] Sharpe LJ, Brown AJ (2013). Controlling cholesterol synthesis beyond 3-hydroxy-3-methylglutaryl-CoA reductase (HMGCR). J Biol Chem.

[17] Sharpe LJ, Coates HW, Brown AJ (2020). Post-translational control of the long and winding road to cholesterol. J Biol Chem.

[18] Crameri A, Biondi E, Kuehnle K, Lutjohann D, Thelen KM, Perga S, Dotti CG, Nitsch RM (2006). The role of seladin-1/DHCR24 in cholesterol biosynthesis, APP processing and Abeta generation in vivo. Embo j.

[19] Bai X, Wu J, Zhang M, Xu Y, Duan L, Yao K, Zhang J, Bo J (2021). DHCR24 knock-down induced tau hyperphosphorylation at Thr181, Ser199, Thr231, Ser262, Ser396 epitopes and inhibition of autophagy by overactivation of GSK3β/mTOR signaling. Front Aging Neurosci.

[20] Greeve I, Hermans-Borgmeyer I, Brellinger C, Kasper D, Gomez-Isla T, Behl C, Levkau B, Nitsch RM (2000). The human DIMINUTO/DWARF1 homolog seladin-1 confers resistance to Alzheimer’s disease-associated neurodegeneration and oxidative stress. J Neurosci.

[21] Thelen KM, Falkai P, Bayer TA, Lütjohann D (2006). Cholesterol synthesis rate in human hippocampus declines with aging. Neurosci Lett.

[22] Kazkayasi I, Ismail MA, Parrado-Fernandez C, Björkhem I, Pekiner C, Uma S, Cedazo-Minguez A, Burul-Bozkurt N (2016). Lack of insulin results in reduced seladin-1 expression in primary cultured neurons and in cerebral cortex of STZ-induced diabetic rats. Neurosci Lett.

[23] Vanmierlo T, Bloks VW, van Vark-van der Zee LC, Rutten K, Kerksiek A, Friedrichs S, Sijbrands E, Steinbusch HW (2010). Alterations in brain cholesterol metabolism in the APPSLxPS1mut mouse, a model for Alzheimer’s disease. J Alzheimers Dis.

[24] Hosseinzadeh S, Zahmatkesh M, Heidari M, Hassanzadeh GR, Karimian M, Sarrafnejad A, Zarrindast MR (2015). Hippocampal DHCR24 down regulation in a rat model of streptozotocin-induced cognitive decline. Neurosci Lett.

[25] Boisvert MM, Erikson GA, Shokhirev MN, Allen NJ (2018). The aging astrocyte transcriptome from multiple regions of the mouse brain. Cell Rep.

[26] Chang TY, Yamauchi Y, Hasan MT, Chang C (2017). Cellular cholesterol homeostasis and Alzheimer’s disease. J Lipid Res.

[27] Orre M, Kamphuis W, Osborn LM, Jansen AHP, Kooijman L, Bossers K, Hol EM (2014). Isolation of glia from Alzheimer’s mice reveals inflammation and dysfunction. Neurobiol Aging.

[28] Lu X, Kambe F, Cao X, Yoshida T, Ohmori S, Murakami K, Kaji T, Ishii T (2006). DHCR24-knockout embryonic fibroblasts are susceptible to serum withdrawal-induced apoptosis because of dysfunction of caveolae and insulin-Akt-Bad signaling. Endocrinology.

[29] Martiskainen H, Paldanius KMA, Natunen T, Takalo M, Marttinen M, Leskelä S, Huber N, Mäkinen P (2017). DHCR24 exerts neuroprotection upon inflammation-induced neuronal death. J Neuroinflammation.

[30] Iivonen S, Hiltunen M, Alafuzoff I, Mannermaa A, Kerokoski P, Puoliväli J, Salminen A, Helisalmi S (2002). Seladin-1 transcription is linked to neuronal degeneration in Alzheimer’s disease. Neuroscience.

[31] Qi Z, Zhang Y, Yao K, Zhang M, Xu Y, Zhang J, Bai X, Zu H (2021). DHCR24 Knockdown lead to hyperphosphorylation of tau at Thr181, Thr231, Ser262, Ser396, and Ser422 sites by membrane lipid-raft dependent PP2A signaling in SH-SY5Y cells. Neurochem Res.

[32] Ariotti N, Fernández-Rojo MA, Zhou Y, Hill MM, Rodkey TL, Inder KL, Tanner LB, Wenk MR (2014). Caveolae regulate the nanoscale organization of the plasma membrane to remotely control Ras signaling. J Cell Biol.

[33] Martin L, Latypova X, Wilson CM, Magnaudeix A, Perrin ML, Yardin C, Terro F (2013). Tau protein kinases: involvement in Alzheimer’s disease. Ageing Res Rev.

[34] Richetin K, Steullet P, Pachoud M, Perbet R, Parietti E, Maheswaran M, Eddarkaoui S, Bégard S (2020). Tau accumulation in astrocytes of the dentate gyrus induces neuronal dysfunction and memory deficits in Alzheimer’s disease. Nat Neurosci.

[35] Alliot F, Pessac B (1984). Astrocytic cell clones derived from established cultures of 8-day postnatal mouse cerebella. Brain Res.

[36] Wilhelm LP, Voilquin L, Kobayashi T, Tomasetto C, Alpy F (2019). Intracellular and plasma membrane cholesterol labeling and quantification using filipin and GFP-D4. Methods Mol Biol.

[37] Amundson DM, Zhou M (1999). Fluorometric method for the enzymatic determination of cholesterol. J Biochem Biophys Methods.

[38] Han T, Lv Y, Wang S, Hu T, Hong H, Fu Z (2019). PPARγ overexpression regulates cholesterol metabolism in human L02 hepatocytes. J Pharmacol Sci.

[39] Sergeant N, Bretteville A, Hamdane M, Caillet-Boudin ML, Grognet P, Bombois S, Blum D, Delacourte A (2008). Biochemistry of Tau in Alzheimer’s disease and related neurological disorders. Expert Rev Proteomics.

[40] Zidovetzki R, Levitan I (2007). Use of cyclodextrins to manipulate plasma membrane cholesterol content: evidence, misconceptions and control strategies. Biochim Biophys Acta.

[41] Luo J, Yang H, Song BL (2020). Mechanisms and regulation of cholesterol homeostasis. Nat Rev Mol Cell Biol.

[42] Chang CC, Lee CY, Chang ET, Cruz JC, Levesque MC, Chang TY (1998). Recombinant acyl-CoA:cholesterol acyltransferase-1 (ACAT-1) purified to essential homogeneity utilizes cholesterol in mixed micelles or in vesicles in a highly cooperative manner. J Biol Chem.

[43] Ramírez-Zacarías JL, Castro-Muñozledo F, Kuri-Harcuch W (1992). Quantitation of adipose conversion and triglycerides by staining intracytoplasmic lipids with Oil red O. Histochemistry.

[44] Cao F, Castrillo A, Tontonoz P, Re F, Byrne GI (2007). Chlamydia pneumoniae–induced macrophage foam cell formation is mediated by Toll-like receptor 2. Infect Immun.

[45] Mulas MF, Maxia A, Dessì S, Mandas A (2014). Cholesterol esterification as a mediator of proliferation of vascular smooth muscle cells and peripheral blood mononuclear cells during atherogenesis. J Vasc Res.

[46] Hill MM, Bastiani M, Luetterforst R, Kirkham M, Kirkham A, Nixon SJ, Walser P, Abankwa D (2008). PTRF-cavin, a conserved cytoplasmic protein required for caveola formation and function. Cell.

[47] Ferrer I, Blanco R, Carmona M, Ribera R, Goutan E, Puig B, Rey MJ, Cardozo A (2001). Phosphorylated map kinase (ERK1, ERK2) expression is associated with early tau deposition in neurones and glial cells, but not with increased nuclear DNA vulnerability and cell death, in Alzheimer disease, Pick’s disease, progressive supranuclear palsy and corticobasal degeneration. Brain Pathol.

[48] Zhu X, Sun Z, Lee HG, Siedlak SL, Perry G, Smith MA (2003). Distribution, levels, and activation of MEK1 in Alzheimer’s disease. J Neurochem.

[49] Pei JJ, Braak H, An WL, Winblad B, Cowburn RF, Iqbal K, Grundke-Iqbal I (2002). Up-regulation of mitogen-activated protein kinases ERK1/2 and MEK1/2 is associated with the progression of neurofibrillary degeneration in Alzheimer’s disease. Brain Res Mol Brain Res.

[50] Favata MF, Horiuchi KY, Manos EJ, Daulerio AJ, Stradley DA, Feeser WS, Van Dyk DE, Pitts WJ (1998). Identification of a novel inhibitor of mitogen-activated protein kinase kinase. J Biol Chem.

[51] Ferrer I, Blanco R, Carmona M, Puig B (2001). Phosphorylated mitogen-activated protein kinase (MAPK/ERK-P), protein kinase of 38 kDa (p38-P), stress-activated protein kinase (SAPK/JNK-P), and calcium/calmodulin-dependent kinase II (CaM kinase II) are differentially expressed in tau deposits in neurons and glial cells in tauopathies. J Neural Transm (Vienna).

[52] Martin L, Latypova X, Wilson CM, Magnaudeix A, Perrin ML, Terro F (2013). Tau protein phosphatases in Alzheimer’s disease: the leading role of PP2A. Ageing Res Rev.

[53] Nolan A, De Paula Franca Resende E, Petersen C, Neylan K, Spina S, Huang E, Seeley W, Miller Z (2019). Astrocytic tau deposition is frequent in typical and atypical Alzheimer disease presentations. J Neuropathol Exp Neurol.

[54] Forman MS, Lal D, Zhang B, Dabir DV, Swanson E, Lee VM, Trojanowski JQ (2005). Transgenic mouse model of tau pathology in astrocytes leading to nervous system degeneration. J Neurosci.

[55] Ferrer I, García MA, González IL, Lucena DD, Villalonga AR, Tech MC, Llorens F, Garcia-Esparcia P (2018). Aging-related tau astrogliopathy (ARTAG): not only tau phosphorylation in astrocytes. Brain Pathol.

[56] Augustinack JC, Schneider A, Mandelkow EM, Hyman BT (2002). Specific tau phosphorylation sites correlate with severity of neuronal cytopathology in Alzheimer’s disease. Acta Neuropathol.

[57] Alonso A, Zaidi T, Novak M, Grundke-Iqbal I, Iqbal K (2001). Hyperphosphorylation induces self-assembly of tau into tangles of paired helical filaments/straight filaments. Proc Natl Acad Sci U S A.

[58] Hanger DP, Anderton BH, Noble W (2009). Tau phosphorylation: the therapeutic challenge for neurodegenerative disease. Trends Mol Med.

[59] Togo T, Dickson DW (2002). Tau accumulation in astrocytes in progressive supranuclear palsy is a degenerative rather than a reactive process. Acta Neuropathol.

[60] Goedert M, Eisenberg DS, Crowther RA (2017). Propagation of tau aggregates and neurodegeneration. Annu Rev Neurosci.

[61] Goetzl EJ, Mustapic M, Kapogiannis D, Eitan E, Lobach IV, Goetzl L, Schwartz JB, Miller BL (2016). Cargo proteins of plasma astrocyte-derived exosomes in Alzheimer’s disease. Faseb j.

[62] Lee EE, Winston-Gray C, Barlow JW, Rissman RA, Jeste DV (2020). Plasma levels of neuron- and astrocyte-derived exosomal amyloid beta1-42, amyloid beta1-40, and phosphorylated tau levels in schizophrenia patients and non-psychiatric comparison subjects: relationships with cognitive functioning and psychopathology. Front Psychiatry.

[63] Resende EPF, Nolan AL, Petersen C, Ehrenberg AJ, Spina S, Allen IE, Rosen HJ, Kramer J (2020). Language and spatial dysfunction in Alzheimer disease with white matter thorn-shaped astrocytes. Neurology.

[64] Howe V, Sharpe LJ, Prabhu AV, Brown AJ (2017). New insights into cellular cholesterol acquisition: promoter analysis of human HMGCR and SQLE, two key control enzymes in cholesterol synthesis. Biochim Biophys Acta Mol Cell Biol Lipids.

[65] Yang T, Espenshade PJ, Wright ME, Yabe D, Gong Y, Aebersold R, Goldstein JL, Brown MS (2002). Crucial step in cholesterol homeostasis: sterols promote binding of SCAP to INSIG-1, a membrane protein that facilitates retention of SREBPs in ER. Cell.

[66] Radhakrishnan A, Goldstein JL, McDonald JG, Brown MS (2008). Switch-like control of SREBP-2 transport triggered by small changes in ER cholesterol: a delicate balance. Cell Metab.

[67] Xu Y, Du X, Turner N, Brown AJ, Yang H (2019). Enhanced acyl-CoA:cholesterol acyltransferase activity increases cholesterol levels on the lipid droplet surface and impairs adipocyte function. J Biol Chem.

[68] Hailstones D, Sleer LS, Parton RG, Stanley KK (1998). Regulation of caveolin and caveolae by cholesterol in MDCK cells. J Lipid Res.

[69] Fielding CJ, Bist A, Fielding PE (1997). Caveolin mRNA levels are up-regulated by free cholesterol and down-regulated by oxysterols in fibroblast monolayers. Proc Natl Acad Sci U S A.

[70] Li S, Song KS, Lisanti MP (1996). Expression and characterization of recombinant caveolin. Purification by polyhistidine tagging and cholesterol-dependent incorporation into defined lipid membranes. J Biol Chem.

[71] Rothberg KG, Heuser JE, Donzell WC, Ying YS, Glenney JR, Anderson RG (1992). Caveolin, a protein component of caveolae membrane coats. Cell.

[72] Fra AM, Williamson E, Simons K, Parton RG (1995). De novo formation of caveolae in lymphocytes by expression of VIP21-caveolin. Proc Natl Acad Sci U S A.

[73] Liu L, Brown D, McKee M, Lebrasseur NK, Yang D, Albrecht KH, Ravid K, Pilch PF (2008). Deletion of Cavin/PTRF causes global loss of caveolae, dyslipidemia, and glucose intolerance. Cell Metab.

[74] Wu C, Butz S, Ying Y, Anderson RG (1997). Tyrosine kinase receptors concentrated in caveolae-like domains from neuronal plasma membrane. J Biol Chem.

[75] Song KS, Li S, Okamoto T, Quilliam LA, Sargiacomo M, Lisanti MP (1996). Co-purification and direct interaction of Ras with caveolin, an integral membrane protein of caveolae microdomains. Detergent-free purification of caveolae microdomains. J Biol Chem.

[76] Parton RG, Hancock JF (2004). Lipid rafts and plasma membrane microorganization: insights from Ras. Trends Cell Biol.

[77] Cai T, Che H, Yao T, Chen Y, Huang C, Zhang W, Du K, Zhang J (2011). Manganese induces tau hyperphosphorylation through the activation of ERK MAPK pathway in PC12 cells. Toxicol Sci.

[78] Lin YT, Cheng JT, Liang LC, Ko CY, Lo YK, Lu PJ (2007). The binding and phosphorylation of Thr231 is critical for tau’s hyperphosphorylation and functional regulation by glycogen synthase kinase 3beta. J Neurochem.

[79] Mahley RW (2016). Central nervous system lipoproteins: ApoE and regulation of cholesterol metabolism. Arterioscler Thromb Vasc Biol.

[80] Nieweg K, Schaller H, Pfrieger FW (2009). Marked differences in cholesterol synthesis between neurons and glial cells from postnatal rats. J Neurochem.

[81] Abad-Rodriguez J, Ledesma MD, Craessaerts K, Perga S, Medina M, Delacourte A, Dingwall C, De Strooper B (2004). Neuronal membrane cholesterol loss enhances amyloid peptide generation. J Cell Biol.

[82] Linetti A, Fratangeli A, Taverna E, Valnegri P, Francolini M, Cappello V, Matteoli M, Passafaro M (2010). Cholesterol reduction impairs exocytosis of synaptic vesicles. J Cell Sci.

[83] Liu Q, Trotter J, Zhang J, Peters MM, Cheng H, Bao J, Han X, Weeber EJ (2010). Neuronal LRP1 knockout in adult mice leads to impaired brain lipid metabolism and progressive, age-dependent synapse loss and neurodegeneration. J Neurosci.

